# Neural architecture in lymphoid organs: Hard‐wired antigen presenting cells and neurite networks in antigen entrance areas

**DOI:** 10.1002/iid3.223

**Published:** 2018-04-10

**Authors:** Clemens Wülfing, Fenja Amrei Schuran, Julia Urban, Jasmin Oehlmann, Hauke Simon Günther

**Affiliations:** ^1^ Group for Interdisciplinary Neurobiology and Immunology Biozentrum Grindel, University of Hamburg Hamburg Germany

**Keywords:** antigen presenting, cell innervation, Lymphoid organ, neurofilaments, neuroimmune crosstalk

## Abstract

**Introduction:**

Recently, we found abundant innervation of antigen presenting cells that were reached and enclosed by single neurites. These neurally hard‐wired antigen presenting cells (wAPC) could be observed in the T‐cell zone of superficial cervical lymph nodes of rats and other mammalians, including humans.

**Methods:**

As a consequence, we investigated lymph nodes at many different anatomical positions as well as all primary and secondary lymphoid organs (SLO) in rodents for a similar morphology of innervation regarding antigen presenting cells known in those tissues.

**Results:**

As a result, we confirmed wAPC in lymph nodes independent from their draining areas and anatomical positions but also in all other T‐cell zones of lymphoid organs, like Peyer's patches, NALT and BALT, as well as in the thymic medulla. Other cells were innervated in a similar fashion but with seemingly missing antigen presenting capacity. Both types of innervated immune cells were observed as being also present in the dermis of the skin. Only in the spleen wAPC could not be detected. Beyond this systematic finding, we also found another regular phenomenon: a dense network of neurites that stained for neurofilament always in antigen entrance areas of lymphoid organs (subsinoidal layer of lymph nodes, subepithelial dome of Peyer's patches, subsinoidal layer of the splenic white pulp, margins of NALT and BALT). Lastly, also thymic epithelial cells (TEC) restricted to the corticomedullary junction of the thymus showed similar neurofilament staining.

**Conclusions:**

Therefore, we propose much more hard‐wired and probably afferent connections between lymphoid organs and the central nervous system than is hitherto known.

## Introduction

The immune and the nervous system are present in nearly all body tissues and organs. And as one main function of both systems seems to be controlling homoeostasis in many directions, one could ask how they control each other. This question and the necessary bi‐directional communication pathways between the immune and the nervous system now engaged researcher interest in many different fields. Besides the long known and well established pathways like the endocrine communication via the neuroendocrine system and the Hypothalamic‐Pituary‐Adrenal‐Axis, much less has been found concerning hard‐wired pathways where neurites innervate lymphoid tissues directly. Today, sympathetic innervation has been shown for most lymphoid organs, whereas the parasympathetic efferents could only be detected in some of these immunologically relevant organs. And looking at the possible afferent pathways, only rudimentary knowledge could be stated with respect to possible autonomic or somatic afferents 1, 2, 3, 4, 5, 6, 7, 8, 9, 10, 11. Lymphoid tissue aggregates integrated in other organs or being organs on their own are divided into primary (PLO) and secondary (SLO) lymphoid organs. Thymus and bone marrow are PLO, whereas the lymph nodes, spleen and Peyer's patches of the intestinal wall are members of the SLO 9, 12, 13, 14, 15, 16, 17, 18, 19, 20, 21, 22, 23. Beside this undisputed classification, other SLO like nasopharynx‐ (NALT) or bronchus associated lymphatic tissue (BALT) vary significantly in different species. In rodents, NALT is most often defined as lymphoid tissue in the floor of the dorsal nasal cavity, while BALT is described as parts of lymphoid tissue all along and inside the bronchial airway walls 24, 25, 26, 27, 28, 29 (see also supplementary Figs. Sa and Sb). In addition to these SLO in the respiratory pathway, Peyer's patches are part of gut associated lymphatic tissue (GALT), beside many other intestinal lymphoid aggregates and diffusely scattered immune cells in the intestine and associated mesentery and omentum 20, 25, 30, 31, 32, 33. NALT, BALT, and GALT are subsumed under the term mucosa associated lymphatic tissue (MALT) separated into inductive or effector tissue 33. Some authors also define parts of the skin as similar to lymphoid organs, which they name skin associated lymphatic tissue (SALT) or skin immune system (SIS) 34, 35, 36, 37. Sometimes complicating it, a clear anatomical nomenclature of different lymph nodes in standard laboratory animals like mice and rats is still missing, and the spleen has major differences in its anatomy when looking at different mammalian species 15, 38, 39, 40, 41, 42. All SLO can function as inductive tissues in initiating an immune response and show division in B‐ and T‐cell zones. Besides this large population of lymphocytes, all lymphoid organs are built by a diverse population of stromal cells forming a reticular network and containing migratory and resident antigen presenting cells (APC, antigen presenting cell(s)). These APC like B‐cells, macrophages, and dendritic cells are characterized by their expression of MHC II. But as clear‐cut this feature is, as difficult is the further characterization or isolation of the latter both cell types: On one hand, macrophages and dendritic cells overall mostly have identical surface markers 15, 43, 44, but on the other hand the same cell type expresses totally different surface markers depending on the species, on the PLO or SLO in which it is located and even on the location in different parenchymal areas of a given SLO 15, 45, 46, 47, 48, 49. Nevertheless, dendritic cells and macrophages express common markers like the integrins CD11b and CD11c, which are part of complement receptors, and also CD68, often part of the lysosomal membrane of macrophages. More restricted, CD103, also an integrin, is often found on dendritic cells in mucosal tissues 48, 50. In some cases, APC seem to be able to also fulfil classical tasks of stromal cells 17, 51, 52, 53 and vice versa 54, 55, 56, 57. As an example marginal zone‐ and marginal metallophilic macrophages in the spleen could be named, that fulfil tasks of stromal cells and that are characterized also by another surface molecule, CD169 17. On the other hand, stromal cells of the thymus, thymic epithelial cells (TEC), are well known for their antigen presenting capacities in negative selection processes 58. Little is known about direct contacts of APC to neuronal structures of the peripheral nervous system in lymphoid organs. One additional element we contributed recently with our finding of “neurally hard‐wired” APC in the T‐cell zone of superficial cervical lymph nodes of rats, which are thousandfold separately innervated as single cells reached by single neurites with a dense neural meshwork covering the cell body but not membranous extensions 59. We discovered that neural connection by staining for neurofilament, a type IV intermediate filament, which is restricted to the cytoskeleton of neuronal tissue, as keratin (type I and II intermediate filament) is restricted to epithelial cells. We also observed dynamic properties of this innervation with MAP2 staining, a protein involved in microtubule assembly during axonal growth and connecting to intermediate filaments. As a consequence, we named those consolidated structure of an APC together with the enclosing neuronal cell process “wired antigen presenting cells” (wAPC). Now in this work, we comprehensibly wanted to know if this innervation morphology of wAPC could be detected in other lymphoid organs.

## Materials and Methods

### Overview of tissues and markers

To get an overview of possible APC innervation in most lymphoid organs, we included inductive SLO that show division in B‐ and T‐cell zones like lymph nodes of different draining areas (superficial cervical, facial, brachial, axillary, superior mesenteric, inguinal, popliteal, and renal nodes), spleen, Peyer's patches, NALT and BALT, because we found the wAPC exclusively located in the T‐cell zones of lymph nodes. T‐cell zones in the different lymphoid tissues were always visualized by CD3 staining. And although there is no clear definable T‐cell zone in the dermis below skin and mucosa we included that tissue, too, as it is located at a similar first place of antigen encounter. Additionally we tested thymus and bone marrow as PLO. As a first step, the tissues were stained for neural structures with neurofilament antibody (SMI 312). In the case that we found innervated cells, we further tested MAP2 for indicating some dynamic properties as well as MHC II for antigen presenting capacities. Due to the fact described above, that APC in part show uniformity and in other parts heterogeneity of marker expression in different lymphoid tissues, we decided to only look for the most common APC markers: CD11b/c and CD103 as dendritic cell integrins, CD68 for macrophages and the signalling molecule CD172a. In the results section, for the sake of clarity, we will concentrate on systematics and outstanding findings; we will not describe every single marker result in detail for every lymphoid tissue (for details see Table 1).

**Table 1:** Overview of the staining result for different markers with wAPC and wIC

	“wAPC” = MHC II+			“wIC” = MHC II−		
	MAP2	CD11b/c/CD103 CD68	CD172a	MAP2	CD11b/c/CD103 CD68	CD172a
Lymph nodes	+	±	+	n.d.		
Peyer's patches	±	±	−	±	±	−
BALT	±	±	−	±	±	−
NALT	±	±	−	±	±	−
Spleen	n.d.			Detected in one case		
Dermis	+	±	−	+	±	−
Thymus	−	−	−	−	−	−

n.d., wired cell‐type was not detectable in that lymphoid organ; “+,” means expressed by the majority of cells; “±,” means some cells express it, some not; “−,” means nearly no cell express it.

### Organs and tissue specimens

Nasopharynx associated lymphatic tissue (NALT) and brain from 8 weeks old female C57BL/6J mice were purchased from Envigo RMS (The Netherlands).

Bronchus associated lymphatic tissue (BALT), thymus, spleen, brain, lymph nodes (superficial cervical, facial, brachial, axillary, superior mesenteric, inguinal, popliteal, and renal nodes), skin, bone marrow and Peyer's patches from the intestine from 12 weeks old female Sprague–Dawley rats were also purchased from Envigo RMS (The Netherlands).

All organs and tissue specimens were removed post mortem and directly snap frozen in liquid Nitrogen.

All experimental procedures were approved by the DEC‐Consult Animal Ethics Committee, Utrecht, The Netherlands, number HAR‐0005 according to all applicable rules, laws and regulations.

For details of species, number of organs, slices and type of sections see supplementary Table S1.

### Immunofluorescence and antibodies

Twenty micrometer frozen sections (Leica CM1850 cryostat/Leica Biosystems Nussloch GmbH, Nussloch, Germany) of all organ and tissue specimens were dried for 20 min. at room temperature and fixed in cold (−20°C) Aceton Methanol (1:1). Blocking of unspecific binding sites was performed by a 5 min incubation (room temperature) with Super‐Block (ScyTek Laboratories, Logan, Utah) and a subsequent washing step for 5 min in PBS (phosphate buffered saline).

Primary antibodies against rat tissue were rabbit anti‐MAP2 (polyclonal) (1:500; SYSY, Göttingen, Germany), mouse anti‐CD11b/c (OX42) (1:500; Abcam plc, Cambridge, UK), mouse anti‐CD103 (OX62) (1:100; Abcam plc, Cambridge, UK), mouse anti‐CD172a (SIRPalpha) or (OX41) (1:100; Abcam plc, Cambridge, UK), mouse anti‐MHC II (OX6) (1:500; Abcam plc, Cambridge, UK), mouse anti‐CD68 (ED1) (1:100; Abcam plc, Cambridge, UK), rabbit anti‐CD3 (SP7), (1:200; DCS, Hamburg, Germany), mouse anti‐CD169 (ED3) (1:50; Bio‐Rad Laboratories GmbH, München, Germany), mouse anti‐Keratin (80) (1:250; Abcam plc, Cambridge, UK), rabbit anti‐LYVE1 (polyclonal) (1:100; Abcam plc, Cambridge, UK), anti‐ mouse‐Peripherin (1:500; Abcam plc, Cambridge, UK) and mouse anti‐NF (SMI312) (1:100; BioLegend, San Diego, CA).

As primary antibodies against mouse tissue we used the same antibodies as listed above for the rat with the exception of anti‐CD11b/c, anti‐CD103 and anti‐CD172a because of species specifities. Instead, we used the following antibodies: mouse anti‐CD11b/c (polyclonal) (1:100; Abcam plc, Cambridge, UK), rabbit anti‐CD103 (polyclonal) (1:100; Abcam plc, Cambridge, UK) and mouse anti‐CD172a (SIRPalpha) (polyclonal) (1:100; Abcam plc, Cambridge, UK).

Primary antibodies were diluted in PBS as described above and incubated for 60 min at room temperature or overnight at 4°C, followed by 15 min incubation of secondary antibodies.

Secondary antibodies used were goat anti‐rabbit‐DyLight 549 (1:700; Biomol, Hamburg, Germany), goat anti‐rabbit‐Alexa 488 (1:700; Biomol, Hamburg, Germany), goat anti‐mouse‐DyLight 549 (1:700; Biomol, Hamburg, Germany) and goat anti‐mouse‐Alexa 488 (1:700; Biomol, Hamburg, Germany).

For multicolor staining, primary antibodies in certain cases were conjugated with: Cy3 (Cy3 Fast Conjugation Kit/Abcam plc, Cambridge, UK), FITC (FITC Fast Conjugation Kit/Abcam plc, Cambridge, UK), HiLyteFluor 647 (AnaTag HiLyte Fluor 647/ANASPEC, Inc., Fremont, CA), and HiLyteFluor 488 (AnaTag HiLyte Fluor 488/ANASPEC, Inc.).

Only in the case of mouse anti‐NF (SMI312) (1:100; BioLegend), we always used the primary antibody conjugated with HiLyteFluor 488 (AnaTag HiLyte Fluor 488/ANASPEC, Inc.).

Slides were mounted using Vectashield Hard Set mounting medium with DAPI (Vector Laboratories, Burlingame, CA).

A Zeiss Axio Imager.M2m microscope (Carl Zeiss, Oberkochen, Germany) with Zen software was used for image collection.

To be sure, that neurofilament antibody SMI312 stains neural structures in the PNS (peripheral nervous system), staining controls were performed with tissue specimens containing distinguishable peripheral nerves (NALT, lymph node and dermis) and brain (for some representative results, see supplementary Figs. Sc–Sg).

**Figure 1:**
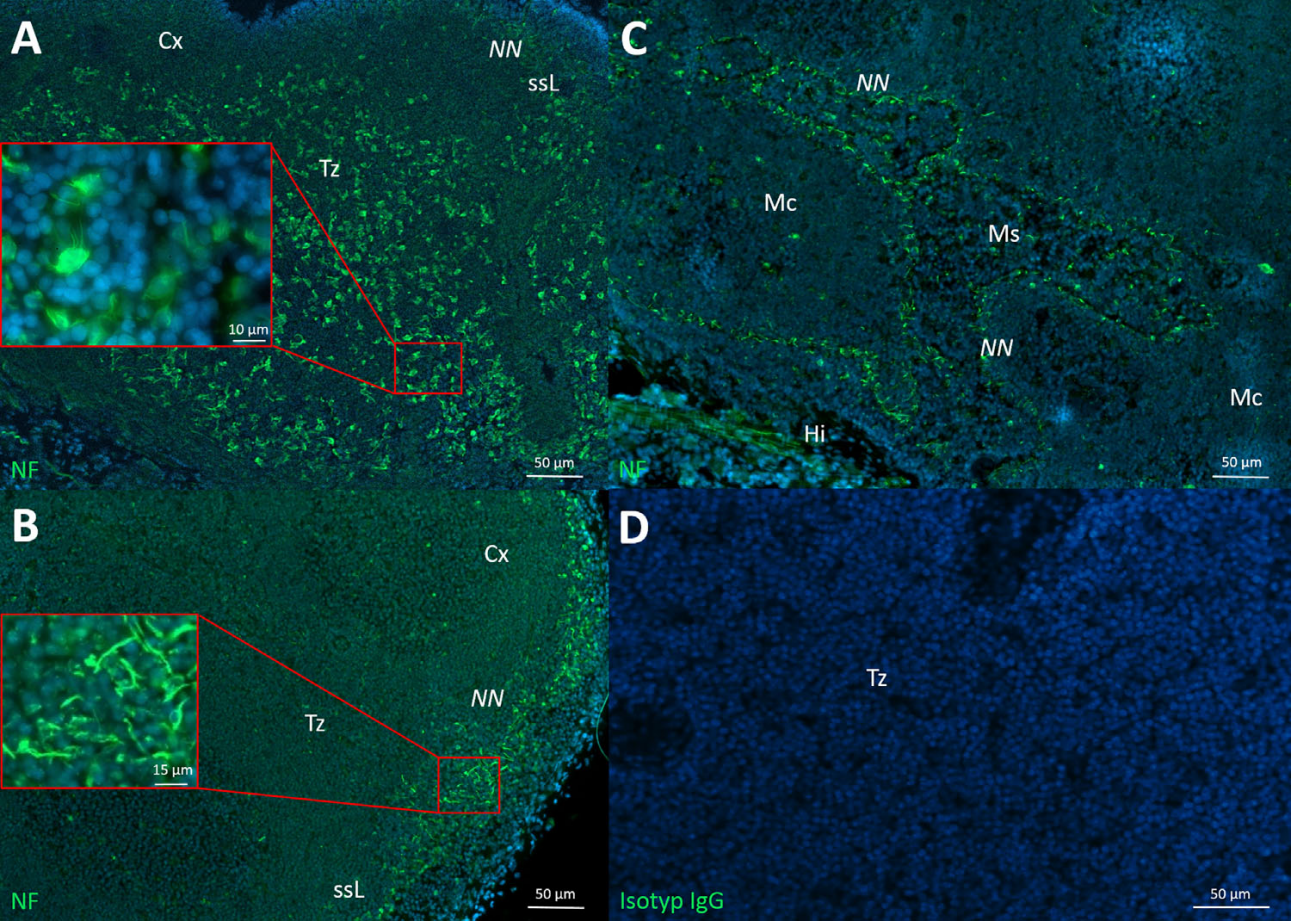
Neural structures and wAPC in different lymph nodes. A: large amounts of neurally hard‐wired antigen presenting cells (wAPC) in the T‐cell zones (Tz) of popliteal lymph nodes, some wAPC are partially enlarged. B: Much lower amounts of wAPC are visible in this renal lymph node; also a prominent neural nexus (NN) can be seen in the subsinoidal layer (ssL) as a dense meshwork of neurites, partially enlarged. C: Neural nexus around all borders of medullary sinuses (Ms) to medullary cords (Mc) in superior mesenteric lymph nodes. D: Isotype control of a popliteal lymph node. (Hi) hilus; (Cx) cortex. Lymph nodes of Sprague–Dawley rats stained with monoclonal anti‐neurofilament (green) and DAPI (blue) in A–C.

**Figure 2:**
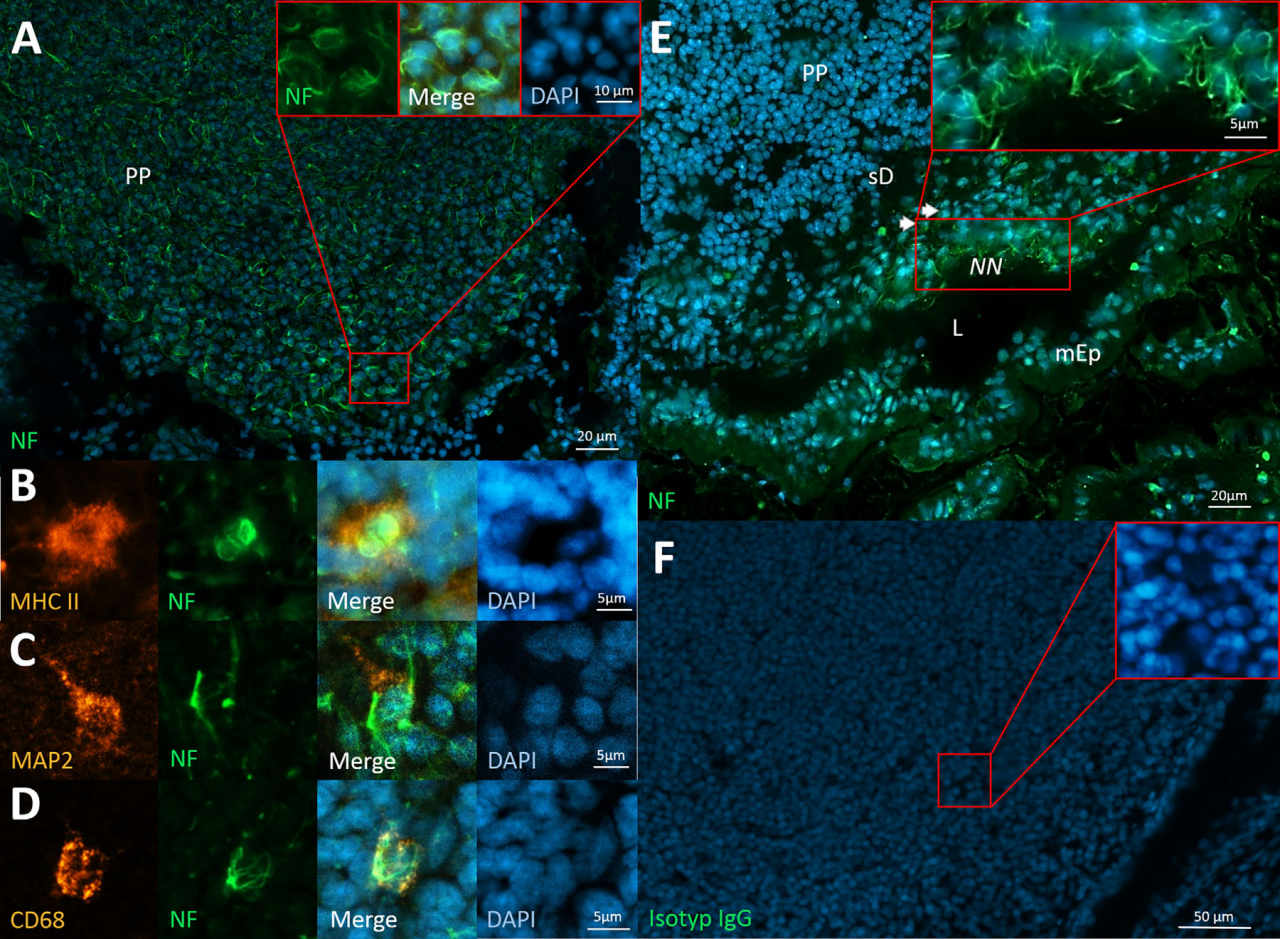
Neural structures in Peyer's patches. A: wAPC innervation patterns in the T‐cell zone of Peyer's patches (PP), partially enlarged. B: wAPC are MHC II positive. C: Only some of the wAPC express MAP2. D: CD68 is rarely expressed by wAPC. E: Neural nexus (NN) appearing at the outer border of a subepithelial dome (sD), partially enlarged; the other mucosa epithel (mEp) surrounding the intestinal lumen (L) is negative for that phenomenon. F: Isotype control of Peyer's patches. Peyer's patches of Sprague–Dawley rats stained with monoclonal anti‐neurofilament (green) and DAPI (blue) in A–E, anti‐MHC II in B (orange), anti‐MAP2 in C (orange)and anti‐CD68 in D (orange).

**Figure 3:**
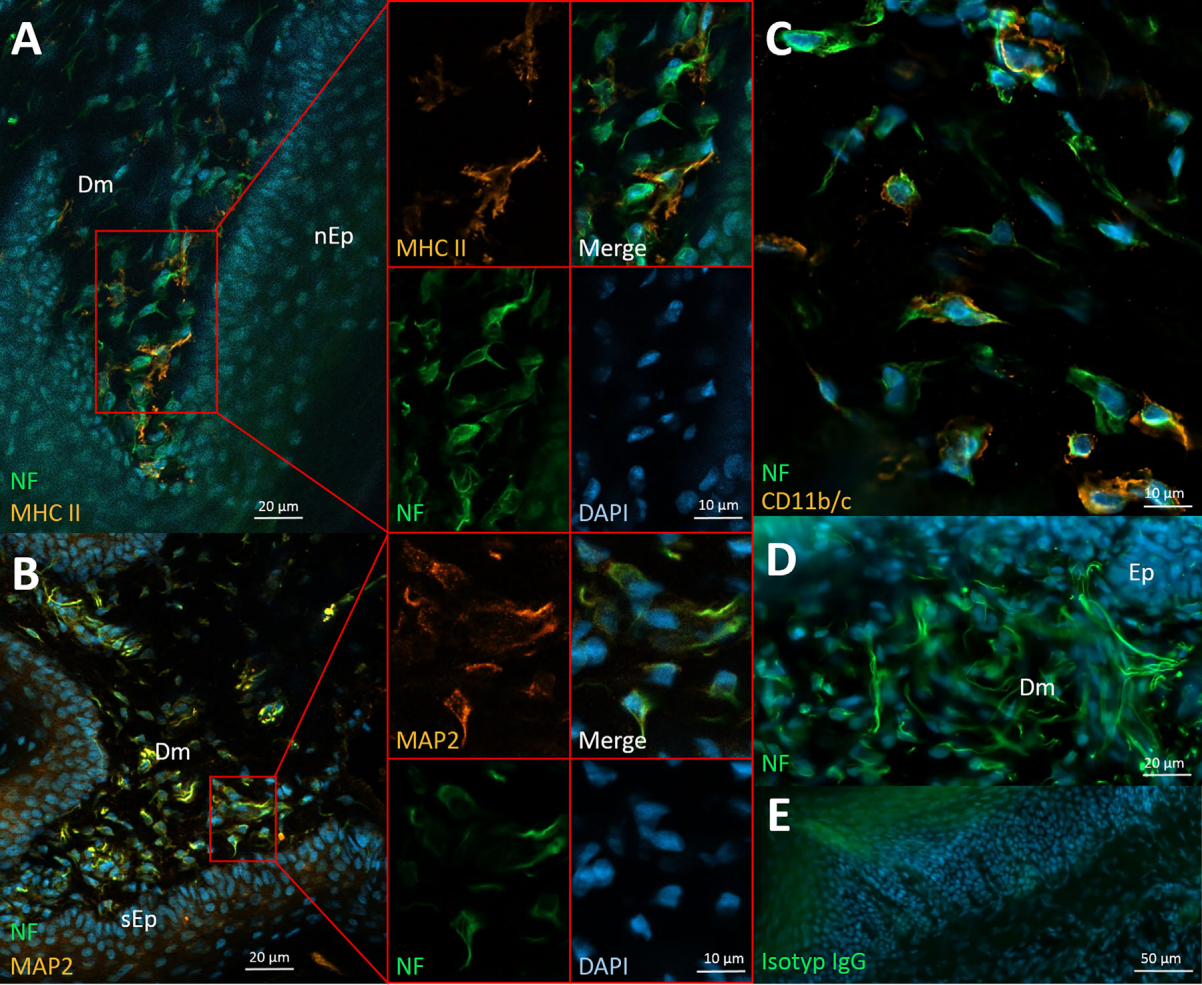
Neural structures of the dermis below skin and mucosa tissue. A: wAPC in the dermis (Dm) below nasal mucosa (nEp) with antigen presenting capacity marked by MHC II expression, side by side with wIC negative for that marker, partially enlarged in split channels. B: Most innervated cells (wAPC and wIC) express MAP2 as is shown in the dermis below skin epithel (sEp). C: Some of the wAPC clearly stained positive for known dendritic cell markers like CD11b/c. D: Many free neurites could be observed in the dermis, with some of them potentially also belonging to a neural nexus (see results section for details). E: isotype control of dermis below mucosa. Dermis of Sprague–Dawley rats stained with monoclonal anti‐neurofilament (green) and DAPI (blue) in A–D, anti‐MHC II in A (orange), anti‐MAP2 in B (orange) and anti‐CD11b/c in C (orange).

**Figure 4:**
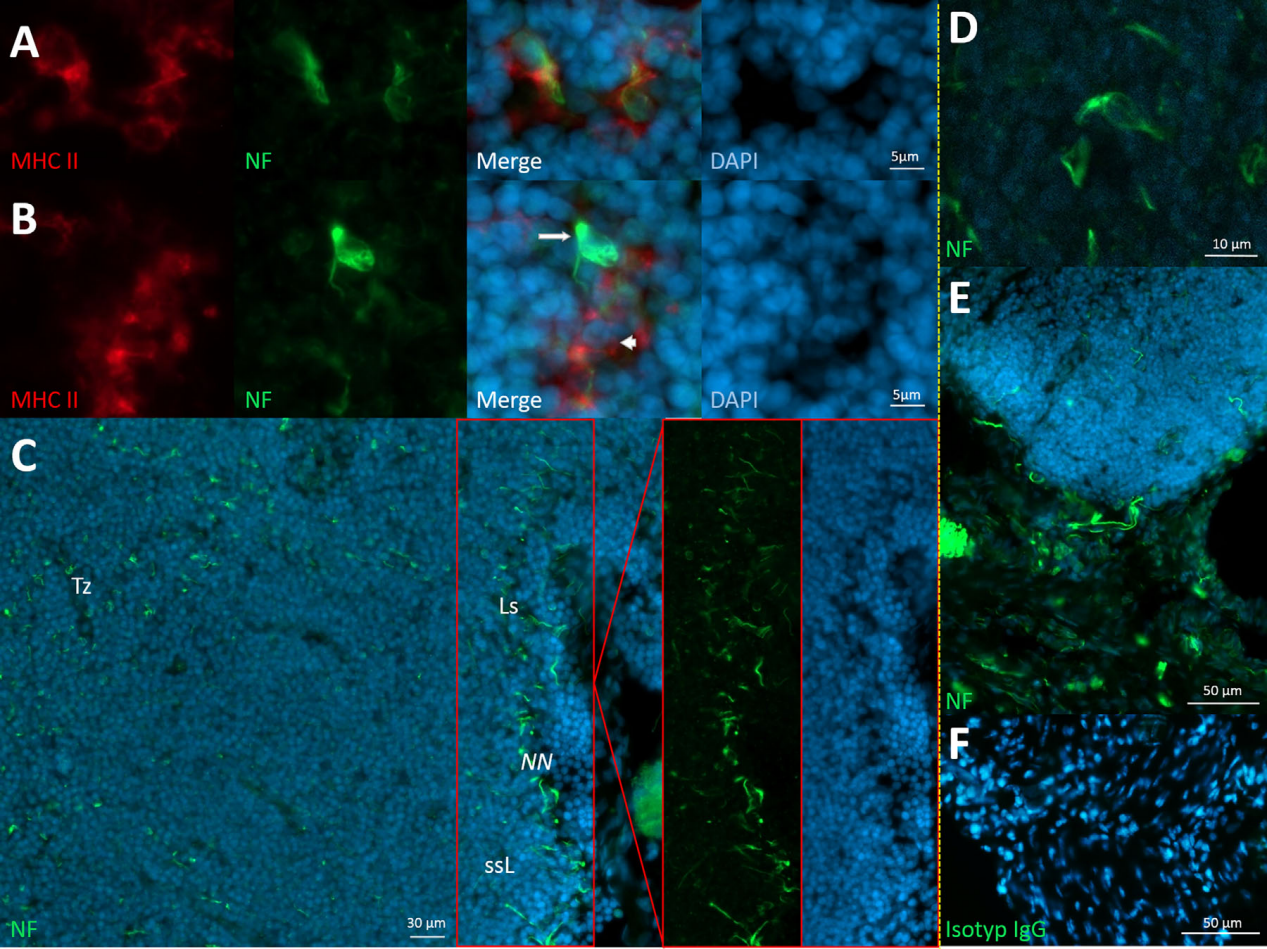
Neural structures of BALT and NALT. A: wAPC in BALT tissue marked by neurofilament and MHC II expression. B: Cells in BALT tissue that either are neurally hard‐wired cells without antigen presenting capacity (wIC)—(arrow), as also cells being APC, but without any demonstrable neural contact (arrowhead). C: Neural nexus (NN) at the border of BALT and wAPC in the T‐cell zone (Tz), with the neural nexus part also shown in split channels to better identify the seemingly subsinoidal layer (ssL) below a lymphatic sinus (Ls) in that area. D: wAPC in NALT tissue. E: Nerves seem to innervate NALT tissue from the periphery. F: Isotype control for NALT tissue. BALT of Sprague–Dawley rats (A–C) and NALT of C57/BL/6 mice (D–F), stained with monoclonal anti‐neurofilament (green) and DAPI (blue) in A–E, anti‐MHC II in A and B (red), with images of both tissue types separated by yellow vertical line.

**Figure 5:**
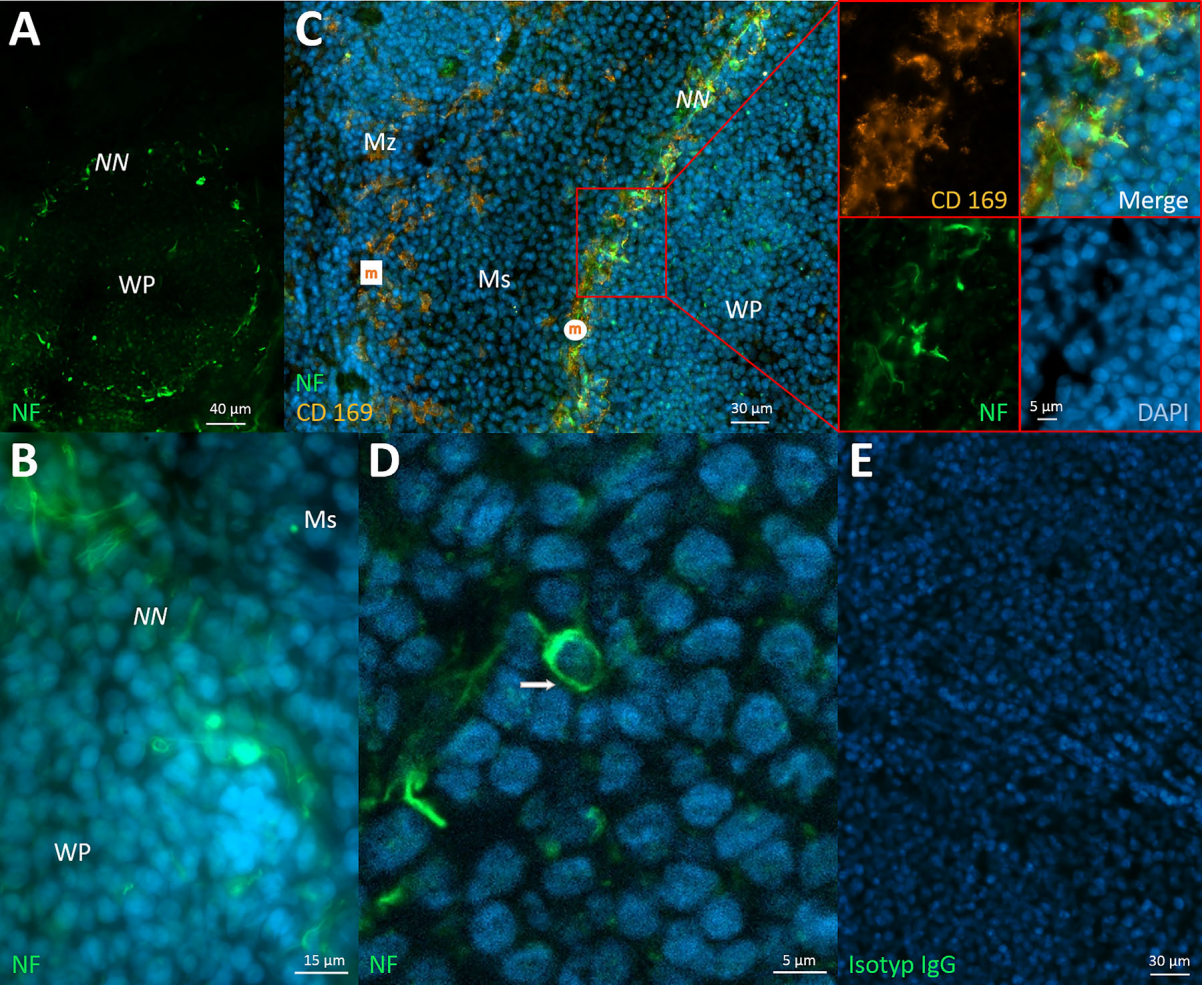
Neural structures in the spleen. A: Neural nexus (NN) surrounding the white pulp (WP) in a spherical shape. B: The fine network of neurites of the neural nexus. C: Staining for CD 169 marked two different macrophage populations—“marginal zone macrophages” (white square named “m”) in the marginal zone (Mz) and “marginal zone metallophilic macrophages” (white circle named “m”) below the marginal sinus (Ms). With it, the exact location of the neural nexus just below the marginal sinus could be specified, and is partially enlarged. D: Attached to the surrounding neural nexus, a wIC (arrow) was found. E: Isotype control for spleen tissue. Spleen of Sprague–Dawley rats stained with monoclonal anti‐neurofilament (green) and DAPI (blue) in A–D and anti‐CD169 in C (orange).

**Figure 6:**
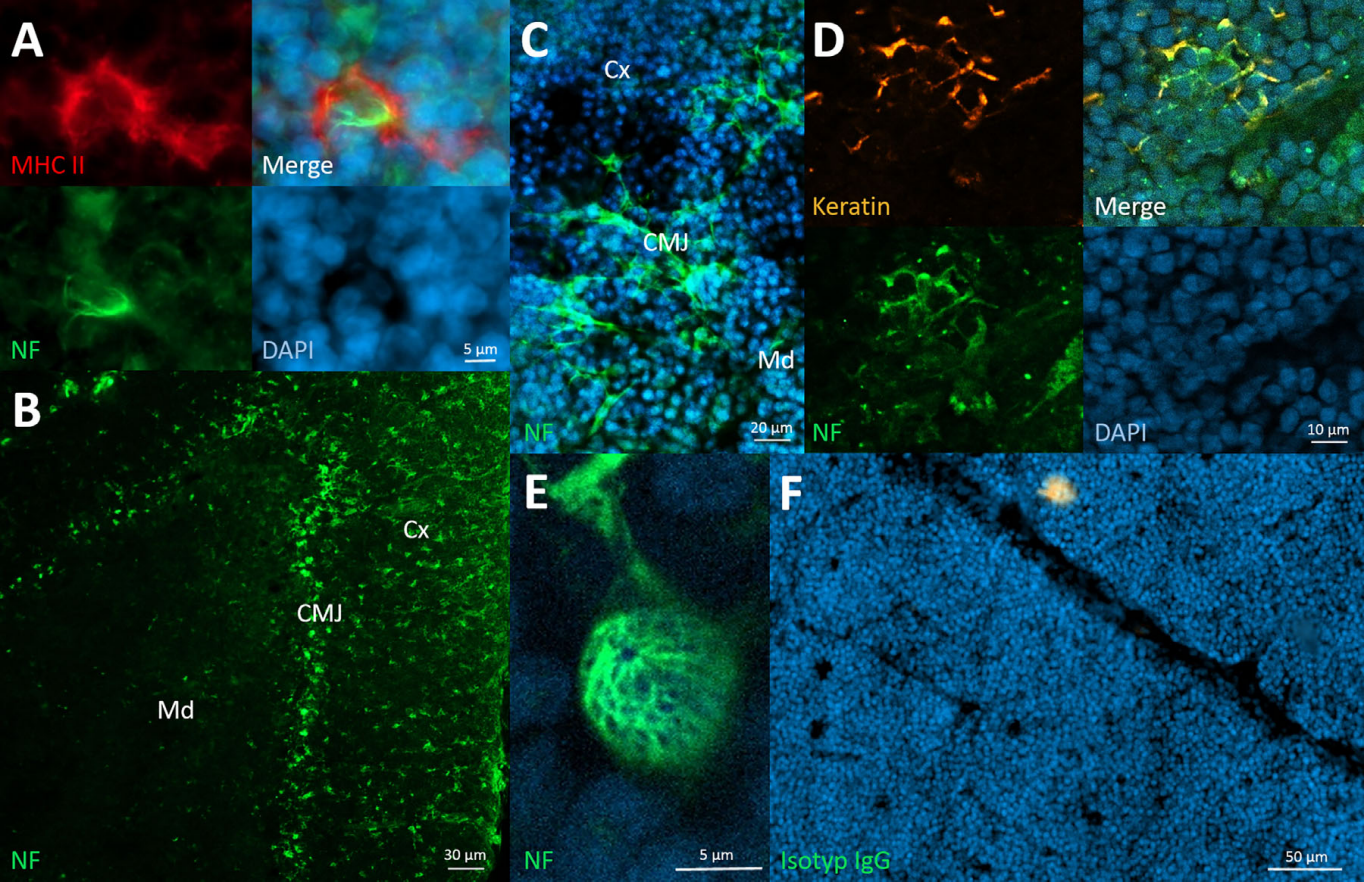
Neural structures in the thymus. A: wAPC in the thymic medulla. B: Neural structures obviously restricted to the corticomedullary junction (CMJ) between cortex (Cx) and medulla (Md) of the thymus. C: Larger magnification revealed neural structures in the corticomedullary junction as a network of cells. D: Keratin staining identified the cells of the neural structures in the corticomedullary junction as TEC, and because of their seemingly simultaneous neurofilament expression we titled them with the prefix “(n)”eural (nTEC). E: TNC closely surrounded by a neural meshwork in the thymic cortex. F: Isotype control of thymic tissue. Thymus of Sprague–Dawley rats stained with monoclonal anti‐neurofilament (green) and DAPI (blue) in A–E, anti‐MHC II in A (red) and anti‐keratin in D (orange).

We performed isotype controls of every used primary immunglobuline (Mouse IgG (MG1‐45) and Mouse IgM (MM‐30); BioLegend, Rabbit IgG (poly); Abcam plc, Cambridge, UK), with every used concentration (specific primary antibody) on every type of tissue with the standard staining protocol. Representative results are shown in Figures 1–6.

To exclude unspecific staining of secondary antibodies, staining procedures were performed without primary antibodies (replaced by PBS) on every single tissue type (Data not shown).

### Decalcification

For frozen bone marrow sections, the femur (rat) was fixed in 4% PFA for 24 h and then placed in 15 ml of 14% EDTA for 2 weeks (solution was refreshed daily). To complete the procedure, tissue was rinsed four times in ddH2O tissue and frozen in Tissue‐Tek® O.C.T.™ Compound (Sakura Finetek, Staufen, Germany) for further processing.

### Hematoxylin and Eosin (H&E) Staining

For histological overviews we stained all tissue specimens with the standard procedure of Hematoxylin and Eosin. 20 μm cryo slides were dried at room temperature, followed by an incubation with Hämalaun (Carl Roth GmbH + Co. KG, Karlsruhe Germany) for 3 min at room temperature. Rinsing in aqua dest. for 1 min and for 10 min in tab water. Eosin (Carl Roth GmbH + Co. KG, Karlsruhe Germany) counterstaining was performed for additional 3 min at room temperature followed by dehydration ascending ethanol series (70–96–100%, 2 × 2 min) and finished with 2 × 2 min incubation in Histol (Carl Roth GmbH + Co. KG, Karlsruhe Germany).

## Results

We recently found hitherto unknown neural structures stained by anti‐neurofilament in lymph nodes of rats like “neurally hard‐wired” APC in the T‐cell zone (wAPC) and dense innervation of the subsinoidal layer. Consequently, we now continued our search for similar neural structures in all other primary and secondary lymphoid organs (SLO). As a result above all, the density of neural structures not associated with blood vessels that could be stained by neurofilament in all lymphoid organs tested was much higher than in any other work we sifted in literature. The complexity of neural architecture in lymphoid organs therefore seems to be much higher than hitherto estimated.

### Lymph nodes—and a puzzling wAPC‐phenomenon

In our last work, we showed and characterized wAPC in superficial cervical lymph nodes of rats, humans and T‐cell deficient mice. Now we checked most other lymph nodes of rats with different draining areas, following a clear anatomical definition 40. As a result, the wAPC could be stained in all selected lymph nodes, with identical morphology, but different amounts. More to the point, the presence of wAPC seemed to be an irregular phenomenon; they were neither they were present in every lymph node of any given type, nor throughout the whole tissue of one lymph node. We often observed dense populations of wAPC in the first draining lymph node of different tissue areas, like skin‐ (popliteal, inguinal) or mucosa‐ (superior mesenteric) draining lymph nodes, which are in the “first front” with APC contact. Much lower amounts of these wAPC have been observed in the downstream‐connected lymph nodes in a “second or third front” along the lymphatic vessel system, like the renal nodes. Confirming our previous results, wAPC always seem to cluster in discontinuous areas below afferent lymphatic vessels with some wAPC also located in interfollicular regions. Another consistent result proved in all lymph nodes investigated was that the highest density of neurites always appeared in the subsinoidal layer right below the subcapsular sinus. Additionally, some lymph nodes showed further structures. In a superior mesenteric and an inguinal lymph node, we observed a similar morphology of a high density of neurites all around the border between medullary sinuses and medullary cords. Interestingly, the latter findings about the dense appearance of neurites was also present in many other lymphoid organs, located in antigen or APC entrance areas. Therefore, we will continue to refer to this phenomenon of a high density of neurites in definable tissue areas as “neural nexus” (Fig. 1).

### Peyer's patches—“hard‐wiring” more immune cells

As expected, the T‐cell zone of this mucosal SLO also contained APC innervated like the wAPC in the lymph node whereas B‐cell follicles were omitted. Showing sometimes deviating innervation morphology, many wAPC seemed to be not fully enclosed by neural structures, appearing as if the projecting neurite was only densely associated with the APC, going over the cell body margins. Moreover, in this lymphoid tissue we also observed cells being innervated, but seemingly without antigen presenting capacity as they missed MHC II expression. Hence, in the following we will use a different term for all other immune cells in lymphoid organs that are reached by neurites and are innervated with a similar morphology like the wAPC, but without showing MHC II expression: “wired immune cell” (wIC). MAP2 expression, which in the lymph nodes is co‐localized with neurofilament expression in the majority of detected wAPC, could only be seen in rare cases of wAPC in the Peyer's patches. On the other hand, macrophage and dendritic cell markers showed a similar heterogeneous distribution along the detected wAPC like in the lymph node. As mentioned before, the top of the subepithelial dome right below the mucosa epithel also showed a neural nexus, the high density of neurites described above for the subsinoidal layer of the lymph node. The normal mucosa epithel without underlying Peyer‘s patches turned out to be negative for this phenomenon (Fig. 2).

### Dermis—Completing lymphatic pathways

As the dermis is not a defined lymphoid organ, a short explanation is required for including it. The previous results for the mucosa‐draining lymphatic pathway, showed the presence of wAPC and a neural nexus in two different types of lymphoid organs in the first and second position to antigen contact (Peyer‘s patches and superior mesenteric lymph nodes respectively). As this was a form of redundant organization, it led us to the suggestion that the same organization could be present in the skin‐draining lymphatic pathway. As we already had positive results regarding the presence of wAPC and a neural nexus for skin draining lymph nodes in a second position to antigen contact, we decided to also check the first position in this pathway—the tissue layers of the skin. We concentrated on the dermis as the corresponding subepithelial connective tissue layer to the lamina propia containing the Peyer‘s patches below the intestinal mucosa epithel. Consistent with all the previous results, we found wAPC in the dermis, showing a similar morphology detected in Peyer‘s patches and lymph nodes as well as also many wIC without any detectable MHC II staining. Most of the wAPC and wIC were also positive for MAP2, indicating some dynamics, whereas dendritic cell markers again were expressed very heterogeneously as always in all examined lymphoid organs. Interestingly, we were also able to stain many single neurites—probably free nerve endings—in the dermis, whereas the previously described sensory nerve fibres in close association to Langerhans cells in the epidermal tissue layer 60 were not detectable through our neurofilament staining (Fig. 3).

### BALT and NALT—Unmasking the neural nexus as a regular phenomenon

Following looming systematics, in both SLO of the respiratory tract (NALT and BALT), wAPC were irregularly present in T‐cell zones, omitting B‐cell areas. Similar to the findings in the Peyer's patches, the innervation morphology of wAPC appeared heterogeneous as some cells were totally enclosed by the neural meshwork, while others seemed to only be partly covered or having just a close association to a surrounding neurite. Also supplementing the results in Peyer‘s patches, BALT and NALT tissues showed many innervated immune cells reached by neural structures similar to the wAPC, but without any demonstrable antigen presenting capacity by MHC II expression—cells we defined above as wIC. At this point it should be emphasized that here like in all other lymphoid organs investigated, of course there were always some APC showing no neural contact at all. Besides this results for innervated single cells, a neural nexus at the margin of BALT and NALT could be confirmed, therefore again appearing at antigen and APC entrance areas. Interestingly, we also observed a zone of lower cell density at the border of BALT, looking like a lymphatic sinus, comparable to the subcapsular sinus of the lymph node or to the marginal sinus in the spleen. And, although we detected this sinus in only a single case, the neural nexus was located exactly inside and below that layer (Fig. 4A–D). LYVE‐1 staining for lymphatic endothel verified the existence of such a lymphatic sinus in BALT tissue. Moreover, staining for the lymphatics offered a new piece of evidence that something like a medullary area for efferent lymphatics exists, seemingly below the T‐cell zone and orientated to the side which is attached to the bronchus (supplementary Fig. Sh). Only for NALT and BALT, but not for any other lymphoid organ investigated, the origin and course of the innervating nerve fibres could be speculated, as we observed bigger nerves close by, which seem to ramify and reach the lymphoid organ from different sides at the periphery (Fig. 4E).

### Spleen—Having an exceptional role

The spleen is responsible for processing blood‐borne antigens, while all other tested SLO so far are involved in lymphatic drainage. Interestingly, just in the spleen no wAPC could be detected in any part of this lymphoid organ. Very different were the observations regarding a neural nexus. Here, the neural nexus presented itself as a hitherto unknown density of single neurites exactly at the border between the red and the white pulp, also irregularly present at different white pulp areas in the tested spleen sections. Extrapolating that network of neurites around a whole white pulp to three dimensions, the neural nexus would build up a structure that forms some kind of hull around the lymphoid organ. With CD169 staining, we proved the location of the neural nexus in more detail, referring to other works and the special rat spleen anatomy 17. CD169 stains two different macrophage populations, marginal zone macrophages in the marginal zone (outer layer “above” the marginal sinus) and marginal zone metallophilic macrophages in the white pulp border (inner layer just “below” the marginal sinus). As a result, the neural nexus seemed to be restricted to the subsinoidal layer exactly below the marginal sinus at the border of the white pulp, therefore resembling the neural nexus positions in lymph nodes, Peyer's patches, BALT and NALT, in being likewise located at the antigen and APC entrance area (Fig. 5A–C). Although we were not able to detect any wAPC in the corresponding T‐cell zones of the white pulp, in one case we noticed a wIC below and connected to the neural nexus (Fig. 5D).

### Thymus and a possible neuro‐epithelial entrance site

After gathering results for all the different SLO, we searched for similar neural structures in association with immune cells in PLO. We found wAPC and other wIC in the thymic medulla, consistently in that area, where much antigen presenting capacity is necessary for negative selection. However, beside the visible neural structures that seem to innervate immune cells with or without antigen presenting capacity, neurofilament staining in the thymus depicted a network of another cell type. Additional keratin staining proved those cells to be thymic epithelial cells (TEC). Interestingly, while keratin marked all types of cortical or medullary TECs throughout the thymus, neurofilament positive TECs are restricted to the corticomedullary junction, the main entrance area for thymocytes. This neurofilament signal—different to the morphology of wAPC or wIC—not only exhibited all the cell bodies, but also followed most of the fine membranous extensions the TEC built up to connect to each other. It was not possible to figure out whether the neurofilament signal was located inside the TEC or outside and only in close association with their cell membrane, as otherwise could be clearly outlined for the wAPC or wIC. Respecting the different cell type as also the different innervation morphology, we will refer to that TEC showing neurofilament signals in the corticomedullary junction as “neural thymic epithelial cell” (nTEC) (Fig. 6A–D). Other TEC related multicellular structures that could be depicted infrequently by neurofilament staining seem to be thymic nurse cell aggregates (TNC), described as structures built of TEC together with thymocytes and other cell types but unknown function 61, 62. Again, the morphology of the neurofilament signal associated with TNC was different from the phenomenon seen with the nTEC at the corticomedullary junction. The TNC aggregates were surrounded by a dense neurofilament network, going over the individual cellular borders with an impression of a gooseberry shape (Fig. 6E), but it also appeared as if associated neurites were bundling in a single fibre, leaving the TNC at a single site.

### Bone marrow—Not seeing does not mean nothing is there

To complete the set of all lymphoid organs, we also tested the other PLO, bone marrow, for similar neural structures. Unfortunately, we were not able to identify any neural structure comparable to our results in all other lymphoid organs thus far. We summarized the findings according to the neural architecture in the different lymphoid organs in Table 2.

**Table 2:** Summary of the neural structures of lymphoid organs in rodents

	“neural nexus” (high density of single neurites probably also containing free neurites in definable tissue areas, that function as a gate) (e.g. antigen entrance areas and/or areas for incoming and/or leaving APC)	“wAPC” and “wIC” (Cells innervated in T‐cell zones with similar innervation morphology shown recently in lymph nodes)	Additional but not consistent findings
Lymph nodes	Yes (subsinoidal layer)	Yes	Neural nexus also at border of medullary cords and sinuses
Peyer's patches	Yes (subepithelial dome)	Yes	
BALT	Yes (margin of lymphoid tissue)	Yes	Sinoidal appearance of the outer border of BALT
NALT	Yes (margin of lymphoid tissue)	Yes	
Dermis	Many free neurites stained as known but yet only non‐immune functions described	Yes	
Spleen	Yes (subsinoidal layer)	No (see add. findings)	One probably lymphoid cell with wIC‐innervation morphology
Thymus	No (see add. findings)	Yes (only in medulla)	“nTEC‐network” at the corticomedullary junction

The location of the neural nexus could most times be specified simply by the cellular distribution of the corresponding area in the lymphoid organ, only in spleen it was confirmed for the marginal zone and marginal sinus through CD169 staining. The identification of the nTEC‐network was done through combined neurofilament and keratin staining of TEC.

## Discussion

The aim of this work was to get an overview of primary and secondary lymphoid organs of Mammalia with respect to the presence of similar innervation morphology of single cells with APC character, recently found in the lymph nodes of rats—wAPC 59. As a result, we not only confirmed the presence of wAPC in most SLO and PLO, but also got a lot more findings regarding abundant, redundant and apparently systematic innervation patterns in all lymphoid organs.

### wAPC and wIC as an abundant innervation pattern in T‐cell zones of SLO

The results that wAPC are present in all lymphoid organs of lymphatic drainage pathways have interesting implications: Migratory APC normally have their first antigen contact just below or inside epithelial tissues like skin or mucosa. Such tissues at “first places” of antigen contact and processing (often named inductive tissues) included in our work have been Peyer‘s patches, BALT and NALT for mucosa and dermis for skin tissue. The corresponding “secondary place,” where migratory APC normally fulfil their antigen presenting tasks are the lymph nodes. Therefore we included superior mesenteric lymph nodes draining mucosa tissues and many other different lymph nodes draining the skin (superficial cervical, inguinal, popliteal etc). Of course, not all lymphatic drainage pathways are already described in a consistent way, as the connection of BALT and NALT to draining lymph nodes is still debated as well as the definition of skin and its underlying dermis as a SLO 34, 35, 36, 37. Nevertheless, with all those tissues, we now covered the whole pathway of antigen entrance, encounter, processing and presenting from both epithelial tissue types, skin and mucosa up to their first draining lymph node. And the results clearly showed that all “first place” mucosa‐ associated SLO tissues (Peyer‘s patches, BALT, NALT) contained wAPC, which cluster in a comparable way in the T‐cell zone, always omitting B‐cell follicles. Perfectly integrating, the same has been confirmed for the other “first place” tissue, the dermis that showed connections to APC directly beneath its epithelial border. But whether those wAPC innervated in the dermis have effector functions, or whether they are wAPC, which recently got their first antigen contact, remains to be evaluated. Anyway, one important consequence of the presence of wAPC in all those inductive tissues is that there seem to be quite earlier nervous connections to APC than those we recently found in lymph nodes. The wAPC innervation patterns seem to be a redundantly and abundantly but irregularly appearing phenomenon in all lymphoid organs of all lymphatic drainage pathways. Consistent with that statement, we found wAPC in all “secondary placed” lymph nodes investigated. Lymph nodes are often serially connected in the lymphatic vessel system, renal lymph nodes are a good example, as they are often the third or fourth lymph node downstream‐connected to the mucosa draining lymphatic pathway starting with the superior mesenteric lymph nodes 38, 39, 40, 42. As already stated, results have been very heterogeneous regarding different lymph nodes, but we often observed high amounts and densities of wAPC in the first lymph nodes of drainage pathways like superficial cervical, inguinal or superior mesenteric lymph nodes. Compared to those lymph nodes, in more downstream‐connected renal nodes wAPC rarely could be observed. Beyond this, wAPC and wIC seem not to be limited to SLO—even earlier, at a time when T‐cells are still in the thymic selection process, the nervous system seems to contact APC. As is known, APC like macrophages and dendritic cells are involved in negative selection in the thymic medulla. These APC could be resident cells but also migrating cells coming in from peripheral tissues, where their origins may be activation by phagocytosis of external antigens 58. So, the appearance of wAPC in the medullary part of the thymus completed the picture of APC innervation throughout the whole antigen recognizing system, even at the earliest checkpoint of T‐cells. But the type and purpose of that neural connection to PLO tissue may differ from that in SLOs in many ways. The deviating results for the SLO taking care of blood‐borne antigens, the spleen, can have different reasons: Either the negative results for the presence of wAPC in the T‐cell zone of the spleen are due to different surface structures because of handling blood instead of lymph, or the reason is a dynamic nature of the wAPC‐innervation, which then may also explain the irregular wAPC appearance in the different lymph nodes and other SLO. We will come back to that dynamic issue in a later section below. Additionally, in some mucosal lymphoid tissues like Peyer's patches, NALT and BALT, many innervated immune cells could be found that were not clearly MHC II positive and which we therefore named wIC. Consequently, we would define wAPC as only one possible subtype of all possible wIC. Interestingly, wAPC and wIC in different lymphoid tissues often showed deviating patterns of the innervation morphology: sometimes the neural structures seem to enclose the whole cell body like could be seen in all investigated lymph nodes and the dermis, whereas in other lymphoid tissues they only partly encover or are simply in close association with immune cells. It remains to be investigated if the reason for that also implies a different form of neural‐immune connection and which different cell types are counting for the wIC. But undoubtedly, this will be of great interest for a more comprehensive understanding of the function of this type of innervation morphology.

### Neural nexus as abundant neural structures in “gate” areas of SLO

The phenomenon of a high density of neurites we first observed in the subsinoidal layer directly below the subcapsular sinus in lymph nodes of rats 59. With this work, we confirmed that phenomenon to be present in all other lymph nodes as well as in all other SLO examined and which we therefore called neural nexus. Deviating from the results for the wAPC, a neural nexus could also be observed in the spleen. Consolidated, we found the neural nexus phenomenon in all SLO, independent of the body compartment they check—lymphatics or blood. And to show the commonalities between the lymphoid organs, we have to look at the localization of all the neural nexus identified: subepithelial dome of Peyer‘s patches, border edges of BALT and NALT, borders between medullary sinuses and cords as well as the subsinoidal layer in lymph nodes and also the subsinoidal layer in the spleen. Summerizing those observations, the neural nexus seems to be always located at tissue areas which functionally are either main antigen or APC entrance areas or exit areas as in the case of the border between medullary sinuses and cords in lymph nodes. Ergo, all of them have their “gate” character in common. With respect to the neural nexus in the spleen, it has to be added that the anatomical features of this organ are very diverse in mammalian species, statements here have to be limited to the animal examined, the rat 15, 17. Nevertheless, the neural nexus surrounding the white pulp in a spatial perception forms a structure like a covering hull. So it helps to illustrate a generally narrow if not gapless covering of the “gates” in other lymphoid organs, as for example, the lymph nodes subsinoidal layer viewed in a spatial context also covers the whole outer border. However, with regards to those neural nexus at “gates” of SLO, how does the dermis match to the results? As we were able to stain many free nerve endings in the dermis, which are positive for neurofilament, many of them of course will count for the well‐described somatic and autonomic sensory purposes. But perhaps some of the huge amounts of these fibres could also be involved in building up a neural nexus, connecting the dermal “gate,” like all the other entrance and exit areas in the different lymphoid organs investigated. And now, having investigated this abundant phenomenon, what, then, is a neural nexus? For now, two possible scenarios are conceivable, both of which can coexist. Either, the neural nexus is built of many free nerve endings innervating the “gate” area, or it contains neurites dividing up from bundled nerves exactly in that area, which then may follow other ways for reaching their final innervation target—which may be a wAPC. At least in one case a sensory role can be assumed: if the neural nexus contains free nerve endings, such innervation usually has receptive functions in the peripheral nervous system. In the other case, if we see so many single neurites on their way for innervation of other targets, it is difficult to propose a functional afferent or efferent role.

### nTEC as neurally connected structure in the “gate” area of PLO

nTEC appeared as neurofilament positiveTEC in the corticomedullary junction of the thymus. And as we have not been able to assign the neurofilament signal exactly to be outside or inside the TEC, two possibilities remain to be most likely: Either the nTEC express neurofilaments as their own intermediate filaments, or the nTEC are accompanied by neural structures, which express those neurofilaments in their neurites, as is the case with the wAPC. Anyway, in both cases the nTEC phenomenon probably will not imply a new type of TEC in general, it rather will outline an additional feature of known cortical TEC or medullary TEC. Looking at the two possibilities in more detail highlights different cellular capabilities that may be linked with them. If nTEC express neurofilaments as intrinsic intermediate filaments, despite the fact that all TEC have epithelial character, these—then more appropriately called “neuroepithelial”—cells may also have neuronal functions. On the other hand, if these nTEC are enclosed with a dense neural meshwork of neuronal origin with the neurofilaments expressed by neurites, we should likely talk about innervated TEC with their known functions, as we have already done with APC and other immune cells in different SLO. In the latter case the difference between the innervation morphology has to be considered: With the nTEC connected to each other, the neurofilament signal follows all those fine membranous extensions, whereas with the wAPC those extensions are always excluded. However, with both possible cases the nTEC phenomenon can be integrated in the looming systematic innervation patterns observed in SLO so far. It is precisely the corticomedullary junction that is described as the main gate for incoming or exiting immune cells like thymocytes and other myeloid cells 12, 13. And exactly here neurofilaments could be stained either in nTEC themselves or in neural structures that are densely associated with the nTEC. Thus, the nTEC network in the thymus parallels the neural nexus phenomenon found in every SLO in a PLO, as this neural structure is also located at the main “gate” for incoming and exiting immune cells.

Unfortunately, little is known about TNC aggregates beside the fact that they also contain TEC and seem to be involved in thymocyte differentiation 58. Nevertheless, the fact that TNC are preferentially located in the thymic cortex points to a possible counterpart role to the wAPC in the thymic medulla. In that theory, the TNC associated and possibly innervating neural structures may fulfil tasks in positive selection in cortical areas whereas the neural structures innervating wAPC may fulfil tasks in negative selection in medullary areas of the thymus.

### Irregular appearance due to different functions, methodical issues, or dynamic innervation?

Taking together the results for the neural structures identified in all lymphoid organs and also in different lymph nodes, one main difference can be observed: Compared to the regular appearance of the neural nexus and nTEC phenomenon, wAPC and wIC appearance, amount and densities were very heterogenous. For this variability, many different reasons are thinkable and may be true side by side. First, there is no exact knowledge about the relation and total amount of APC populations in different lymphoid organs like in different lymph nodes, so the result could simply portray different amounts of APC in total. Second, regarding different lymph nodes, most APC that present antigens there may do this successfully in the first draining nodes. Consequently, if wAPC innervation is a neuro‐immune connection involved in that task, less innervation because of surveillance is necessary in lymph nodes that are only the third or fourth station in lymphatic circulation. The same aspect could explain the probable absence of wAPC in the spleen handling blood‐borne antigens. As blood has a privileged role in being thoroughly separated from pathogens, less wAPC innervation may be necessary. Third, the negative result for wAPC innervation only in the spleen also follows our previously suggested function 59: As every SLO of lymphatic drainage pathways drains a specific area of the body, the wAPC may be a sensory neuro‐immune connection with topographical content, informing the nervous system about the place where the APC encountered the antigen by assigning signals from different SLO to specific body areas. Such a type of information from the spleen would not make sense, as blood flows irregularly through the whole circulation, ergo a topographical wAPC system would be needless. The fourth possible reason is a methodical issue, the limits of antibody staining. Already using a pan‐neurofilament antibody SMI 312, it cannot be excluded that more neural structures exist that are not visualized. This suggestion is supported by initial studies of our lab, revealing that staining for the single neurofilament chains do not parallel the results of the pan‐neurofilament antibody. It becomes even more probable looking at the vast possibilities for epitopes due to phosphorylation at numerous different sites of the tail region of this type IV intermediate filament 63. The fifth possible reason returns to the dynamic issue: If the wAPC‐innervation belongs to a dynamic system, we would have always observed snapshots of different physiological or pathophysiological processes, depending on the state “frozen” at the time we prepared the tissue. Besides many other dynamic immune processes, this could implicate that wAPC innervation only appears if antigen contact with external antigens has been established, and that the innervated APC in the SLO are of a mostly migratory origin. As a consequence, the wAPC would at least partly belong to a population of temporarily “hard‐wired” APC that get dynamically established neural connections. The results for MAP2 staining supported this theory. Nevertheless, it cannot be excluded that wAPC like wIC are also in part resident cells of lymphoid organs with a permanently “hard‐wired” neural connection. Such dynamic properties of the innervation may also explain other non‐consistent findings in Table 2, like a neural nexus at the border of medullary sinus and cords in lymph nodes.

### Nervous system surveillance

Obviously, looking at our results, the many different places of the whole antigen recognizing system seem not to be blind spots of the nervous system. All places show neural structures in a form of systematic and redundant organization in all lymphoid organs. Hereby, two phenomena can be differentiated, first the similar but irregular appearing innervation patterns of wAPC and wIC in T‐cell zones, and second a neural nexus in “gate” areas of lymphoid organs (including nTEC as the thymic counterpart). Summarizing this together with unpublished results regarding the afferent nature of this connection, an intriguing view of a sensory network arises:

The whole immune response chain, from the early T‐cell selection processes in the thymus throughout the places of innate and adaptive responses up to effector regions, may be under dense surveillance of the central nervous system. This surveillance, then, seems to be concentrated on the one hand at the place of antigen processing or antigen presenting (T‐cell zones of lymph nodes, Peyer's patches, NALT, BALT but also thymic medulla), with all that tissues systematically showing the innervation morphology of wAPC. On the other hand, neural surveillance seems to be concentrated at the “gates” for antigen or APC entrance (subsinoidal layer of lymph nodes and spleen, subepithelial dome of Peyer‘s patches, margins of BALT and NALT), with each systematically showing the phenomenon of a neural nexus most likely containing many free neurites. Moreover, even exit places seem to be included (medullary sinuses to cord border). In this scheme, at least some of the many free nerve fibres in the dermis may likewise function for sensory purposes in the surveillance of the immune system, resembling the neural nexus of “true” lymphoid organs. Anyway, even if we strongly suggest those neural structures to be of an afferent nature, the other efferent possibility has to be considered. But however, afferent or efferent, the fact that all those neural structures can be stained with the same neurofilament antibody points to a common function accompanied by similar cytoskeletal structures.

### Next steps—Bone marrow and further evidence

Preparation of bone marrow as whole slices is always a challenging task because of being able to cut the tissue after treating osseous structures without destroying the sensitive marrow tissue 14. We strongly believe that the negative result we got may inhere in two things. First, the treatment of the tissue as previously described may simply result in the destruction of fine nerve fibres. Second, the recently described clusters of APC together with lymphocytes, which seem to be able to initiate adaptive immune responses like SLO, are very small and only far flung through the bone marrow 64, so they probably escaped our analyses. Nevertheless, we are convinced that more detailed research on this important tissue is necessary with regard to terms of wAPC, wIC, or the presence of a neural nexus, especially in the above‐mentioned clusters for adaptive immune responses.

Finally, many questions remain:

What type of immune cells are innervated in the different lymphoid organs?

To get first evidence, all those innervated cells in the different lymphoid organs have to be checked for their expression profiles. Indeed this will be a mammoth task to accomplish. Not only do all lymphoid organs, by issues of their tissue architecture, differ in preparation methods, but also the correct markers for antigen presenting cells will differ from species to species, from organ to organ and even from parts of the parenchymal tissue.

What is the origin of the innervating neurites and how do they “hard‐wire”?

Only knowing the origin of the innervating neurites together with functional experiments will give a reliable answer to the afferent or efferent nature of the neural structures. It is also important to state that in a strictly neurobiological interpretation, innervation as an expression can only be used if synaptic vesicular transmission has been shown. And as we already searched for that transmission possibility but were not able to find any hint in recent work 59, alternative communication channels, like long‐known electric synapses and also adhesion molecules have to be considered for building those neuro‐immune connections.

With more answers to these questions it may be that not only an “immune sense” will be confirmed, but also an “immune consciousness” will be unravelled.

## Conclusion

We recently identified innervated APC in the T‐cell zone of lymph nodes of rats. These wAPC thousandfold showed an innervation morphology as single cells, separately reached by single neurites and hard‐wired with a dense neural meshwork covering the cell body but not membranous extensions. Here, we continued our recent work in the search for those neurally hard‐wired connections of the nervous system to APC in lymphoid organs.

First, we confirmed the presence of wAPC in all other lymph nodes at different anatomical locations, like also in all other secondary lymphoid organs except the spleen, but also in the dermis of skin and mucosa and in a primary lymphoid organ (PLO), the thymus.

Second, in lymphoid organs and other tissues with first antigen contact, like Peyer's patches, NALT and BALT but also thymic medulla and dermis, we found likewise innervated cells but without clear MHC II expression—wIC. This wIC, together with the wAPC in these tissues, often showed deviating innervation patterns compared to the neural meshwork in the lymph nodes, but all have in common a very close association of the innervating neural structure to only the cell membrane of the cell body, not following membranous extensions.

Third, we showed that most known “gates” of the immune system, no matter if for antigens or APC, for entrance or exit—like the subsinoidal layer of the lymph nodes and spleen, the subepithelial dome of Peyer's patches, the margins of NALT and BALT, and probably also the dermis—stained for a high density of single neurites, a phenomenon we called neural nexus and which we suggest contains many free nerve endings.

Fourth, in the thymus there seem to be an also hitherto unknown staining of neurofilaments closely associated with thymic epithelial cells and restricted to the corticomedullary junction, which leads us to name these cells nTEC. Whether nTEC express neurofilaments in an intrinsic fashion, or if their membranes are closely covered by neurites could not be resolved.

wAPC and wIC, therefore, should define an abundant innervation morphology of probably many different antigen presenting cells and other cells of the immune system, possibly including stromal cells with all their differing expression profiles, whereas neural nexus should characterize a dense network of neurites thought to contain also free nerve endings at most, if not all, “gates” of the lymphoid organs.

For the aforementioned reasons, and actual research with unpublished results, we strongly suggest wAPC and wIC, neural nexus and nTEC to be part of an afferent and sensory system for the surveillance of the immune system.

The functional aspect of this fascinating system distributed through the whole immune system of mammalia awaits further discoveries and is central to our lab's research.

## Conflict of Interest

This research did not receive any specific grant from funding agencies in the public, commercial, or not‐for‐profit sectors.

## Supporting information

Additional supporting information may be found in the online version of this article at the publisher's web‐site.

**Figure Sa**. A: Location of BALT tissue (arrows) in the bronchial airway walls, associated with some alveolar tissue (Av) and preferentially located at bronchial (Br) bifurcations.
**Figure Sb**. A: Position of two NALT aggregates marked by arrows in the floor of the dorsal nasal cavity below the stratified squamous epithel (Ep) and the dermis (Dm).
**Figure Sc**. A to D: Counterstaining with anti‐peripherin, a marker for peripheral nerves and anti‐neurofilament clearly shows a partial overlap but also a partial co‐staining of both markers in a peripheral nerve in the hilus region of a superficial cervical lymph node.
**Figure Sd**. The typically beaded appearance of a huge peripheral nerve passing nearby an axillary lymph node can be seen. Lymph nodes of Sprague–Dawley rats stained with monoclonal anti‐neurofilament (green) and DAPI (blue).
**Figure Se**. The typically beaded appearance of peripheral nerves drawing through the palatal area and surrounding the NALT can be observed. NALT (white arrow) surrounded by the palatal area of C57/BL/6 mice stained with monoclonal anti‐neurofilament (green) and DAPI (blue).
**Figure Sf**. A to D: Counterstaining with anti‐MAP2 and anti‐neurofilament demonstrates crossing and long axonal fibres of peripheral nerves in the palatal area which are double positive for both markers. Dermis below nasal mucosa of Sprague–Dawley rats stained with monoclonal anti‐neurofilament (green), anti‐MAP2 (orange) and DAPI (blue).
**Figure Sg**. Positive control for neurofilament staining in the brain. Brain of Sprague–Dawley rats stained with monoclonal anti‐neurofilament (green) and DAPI (blue).
**Figure Sh**. A: With CD3 staining, BALT tissue can be clearly divided in T‐cell (Tz) and B‐cell (Bz) areas. (Av) Alveolar tissue.
**Table S1**. Statistical information about species, number of organs, slices and type of section.
