# Supplementary material for: Neural architecture in lymphoid organs: Hard‐wired antigen presenting cells and neurite networks in antigen entrance areas
Source: Immun Inflamm Dis. 2018 Apr 10;6(2):354–70. doi: 10.1002/iid3.223 (PMC5946157; doi:10.1002/iid3.223)
Supplement: Supplementary file 1 — Figure Sa. A: Location of BALT tissue (arrows) in the bronchial airway walls, associated with some alveolar tissue (Av) and preferentially located at bronchial (Br) bifurcations. Figure Sb. A: Position of two NALT aggregates marked by arrows in the floor of the dorsal nasal cavity below the stratified squamous epithel (Ep) and the dermis (Dm). Figure Sc. A to D: Counterstaining with anti‐peripherin, a marker for peripheral nerves and anti‐neurofilament clearly shows a partial overlap but also a partial co‐staining of both markers in a peripheral nerve in the hilus region of a superficial cervical lymph node. Figure Sd. The typically beaded appearance of a huge peripheral nerve passing nearby an axillary lymph node can be seen. Lymph nodes of Sprague–Dawley rats stained with monoclonal anti‐neurofilament (green) and DAPI (blue). Figure Se. The typically beaded appearance of peripheral nerves drawing through the palatal area and surrounding the NALT can be observed. NALT (white arrow) surrounded by the palatal area of C57/BL/6 mice stained with monoclonal anti‐neurofilament (green) and DAPI (blue). Figure Sf. A to D: Counterstaining with anti‐MAP2 and anti‐neurofilament demonstrates crossing and long axonal fibres of peripheral nerves in the palatal area which are double positive for both markers. Dermis below nasal mucosa of Sprague–Dawley rats stained with monoclonal anti‐neurofilament (green), anti‐MAP2 (orange) and DAPI (blue). Figure Sg. Positive control for neurofilament staining in the brain. Brain of Sprague–Dawley rats stained with monoclonal anti‐neurofilament (green) and DAPI (blue). Figure Sh. A: With CD3 staining, BALT tissue can be clearly divided in T‐cell (Tz) and B‐cell (Bz) areas. (Av) Alveolar tissue. Table S1. Statistical information about species, number of organs, slices and type of section. [file IID3-6-354-s001.pdf]

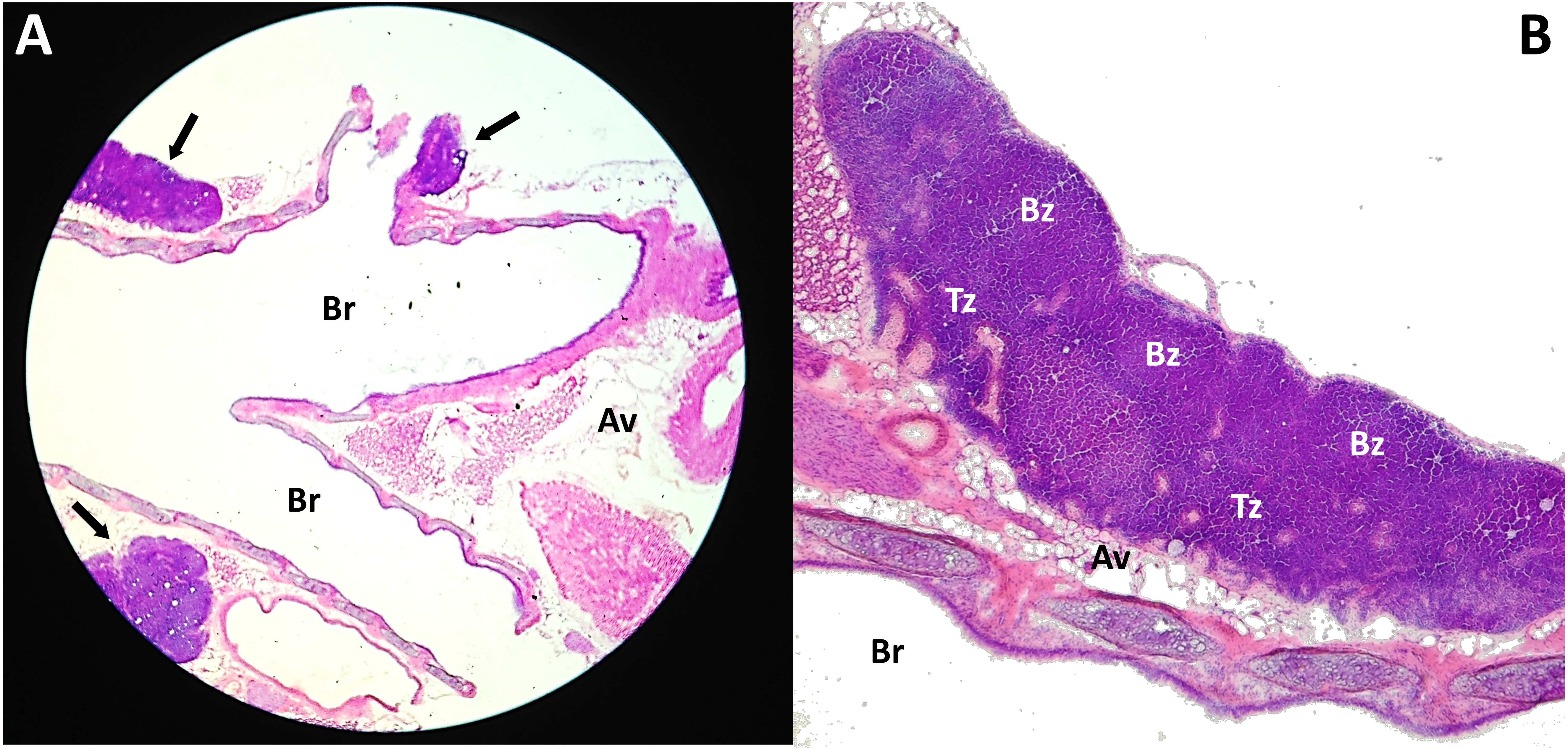

Supplementary figure a (BALT)

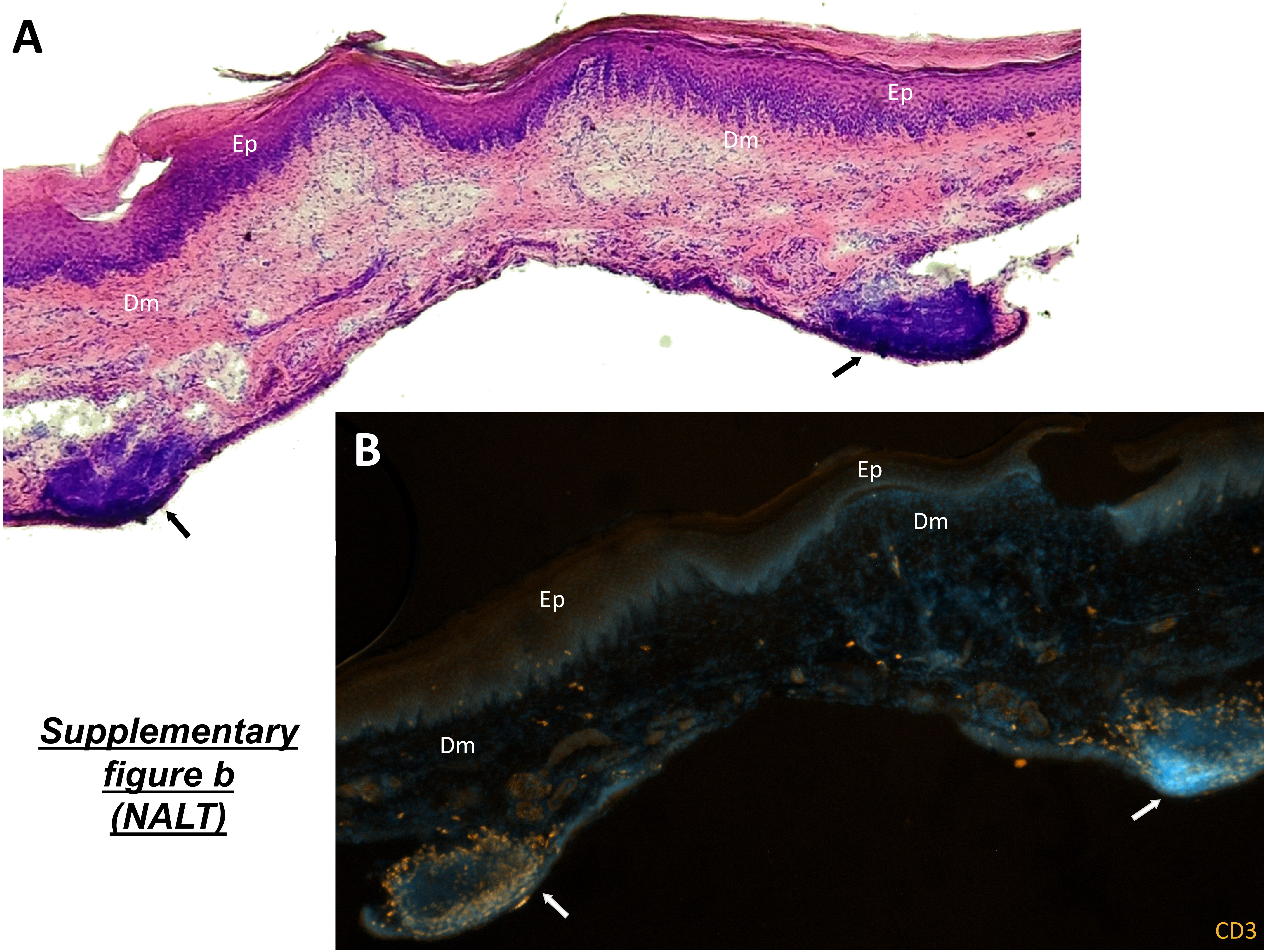

Supplementary figure b (NALT)

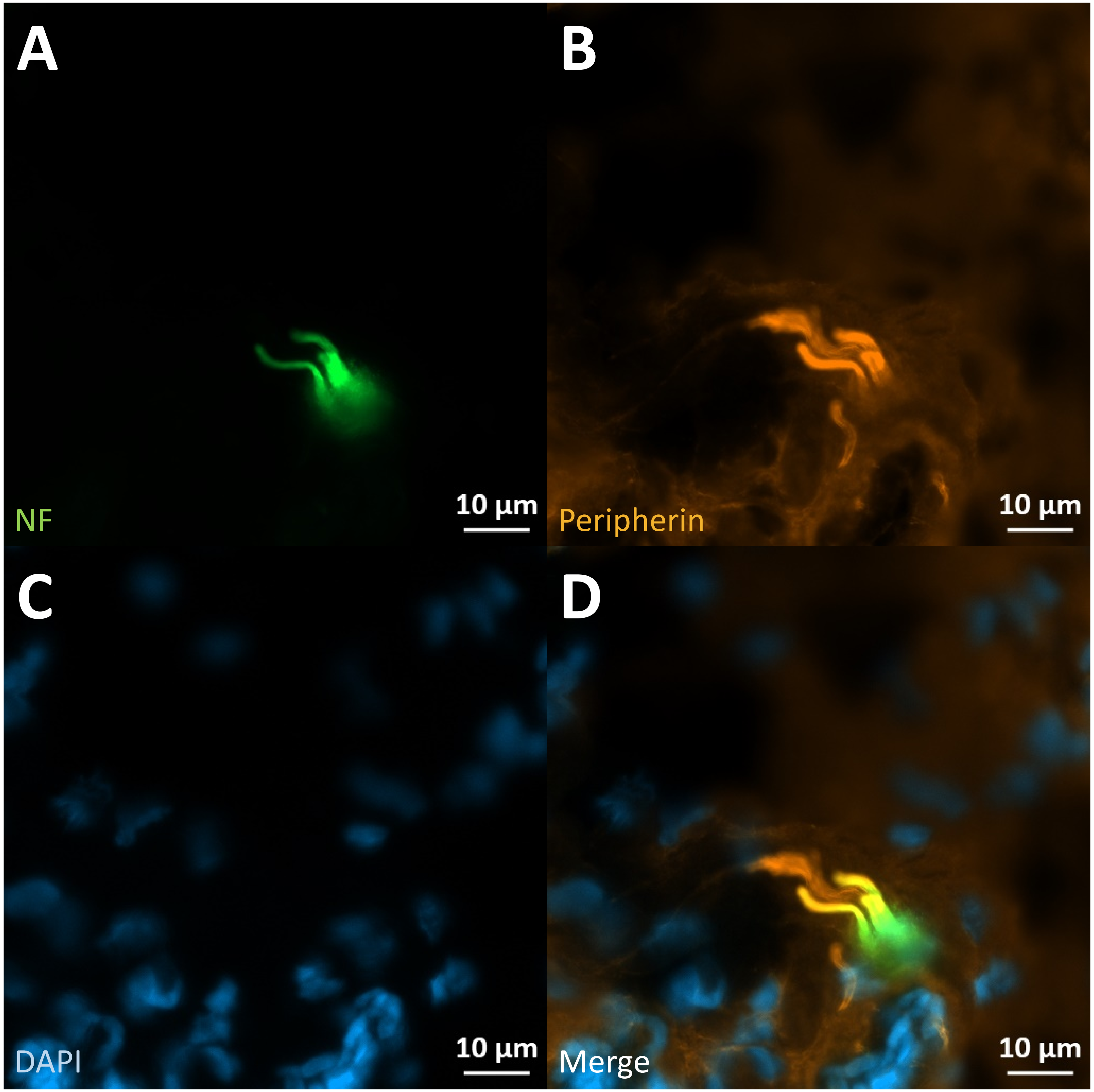

Supplementary figure c

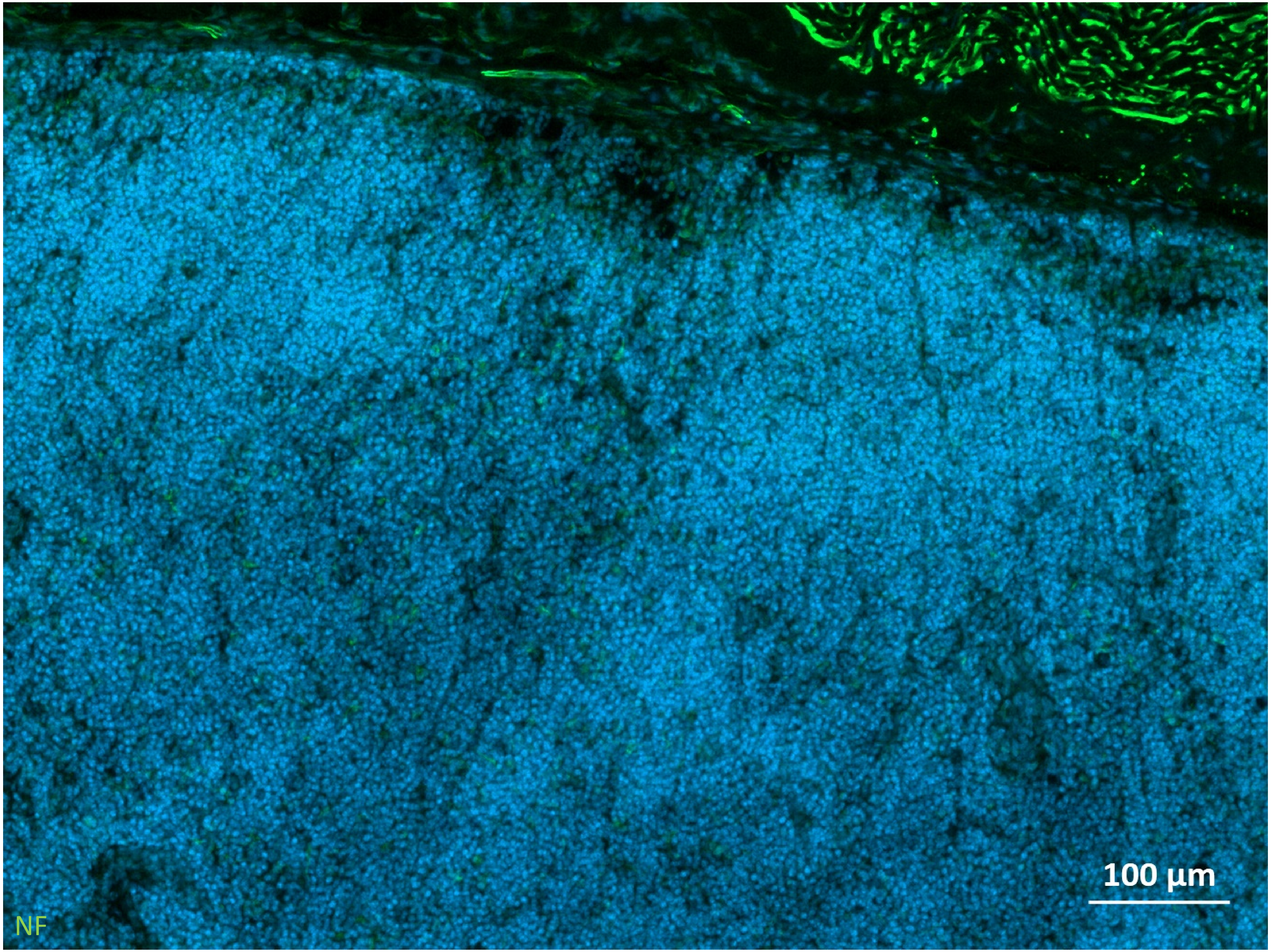

Supplementary figure d

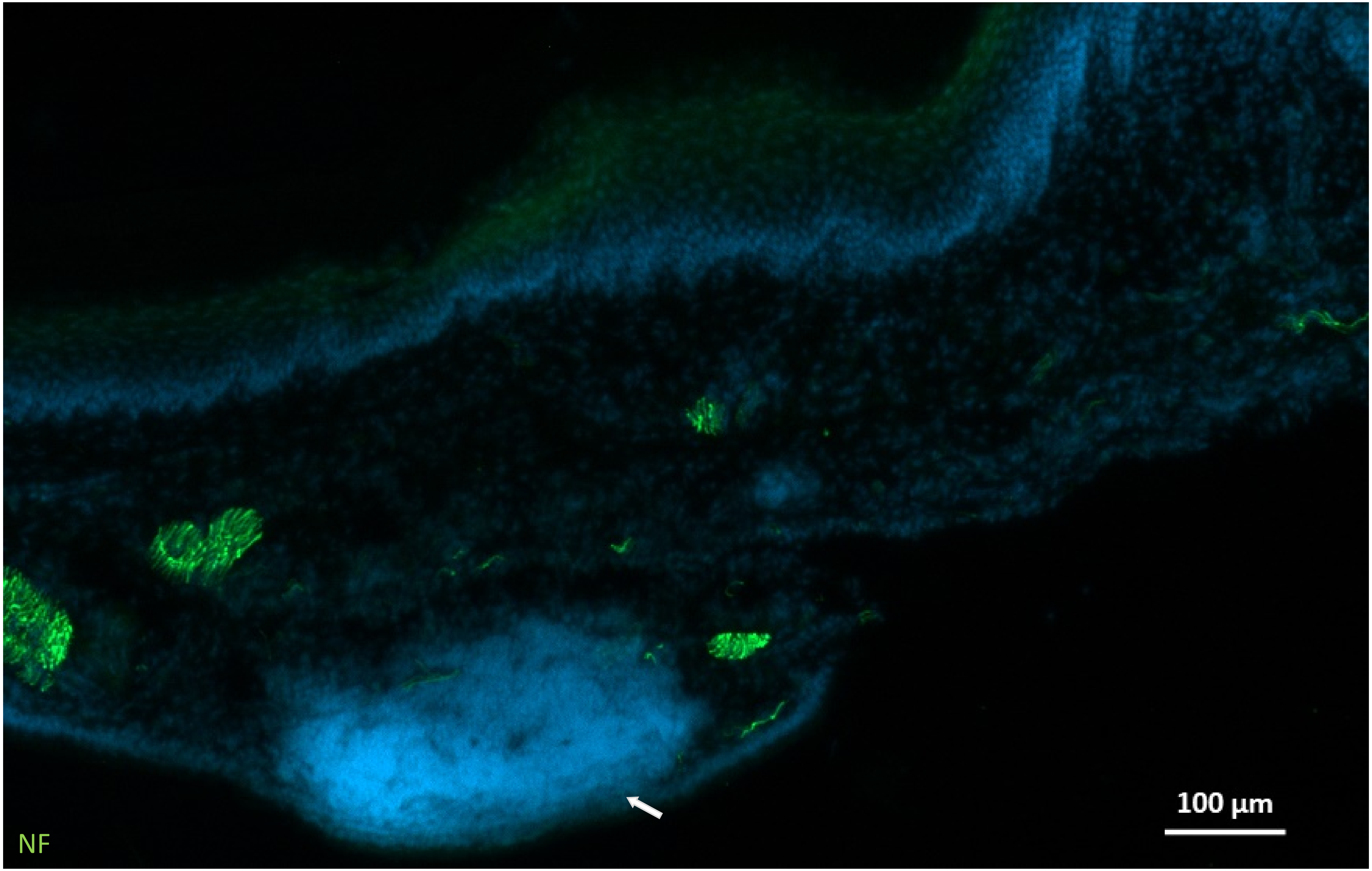

<u>***Supplementary figure e***</u>

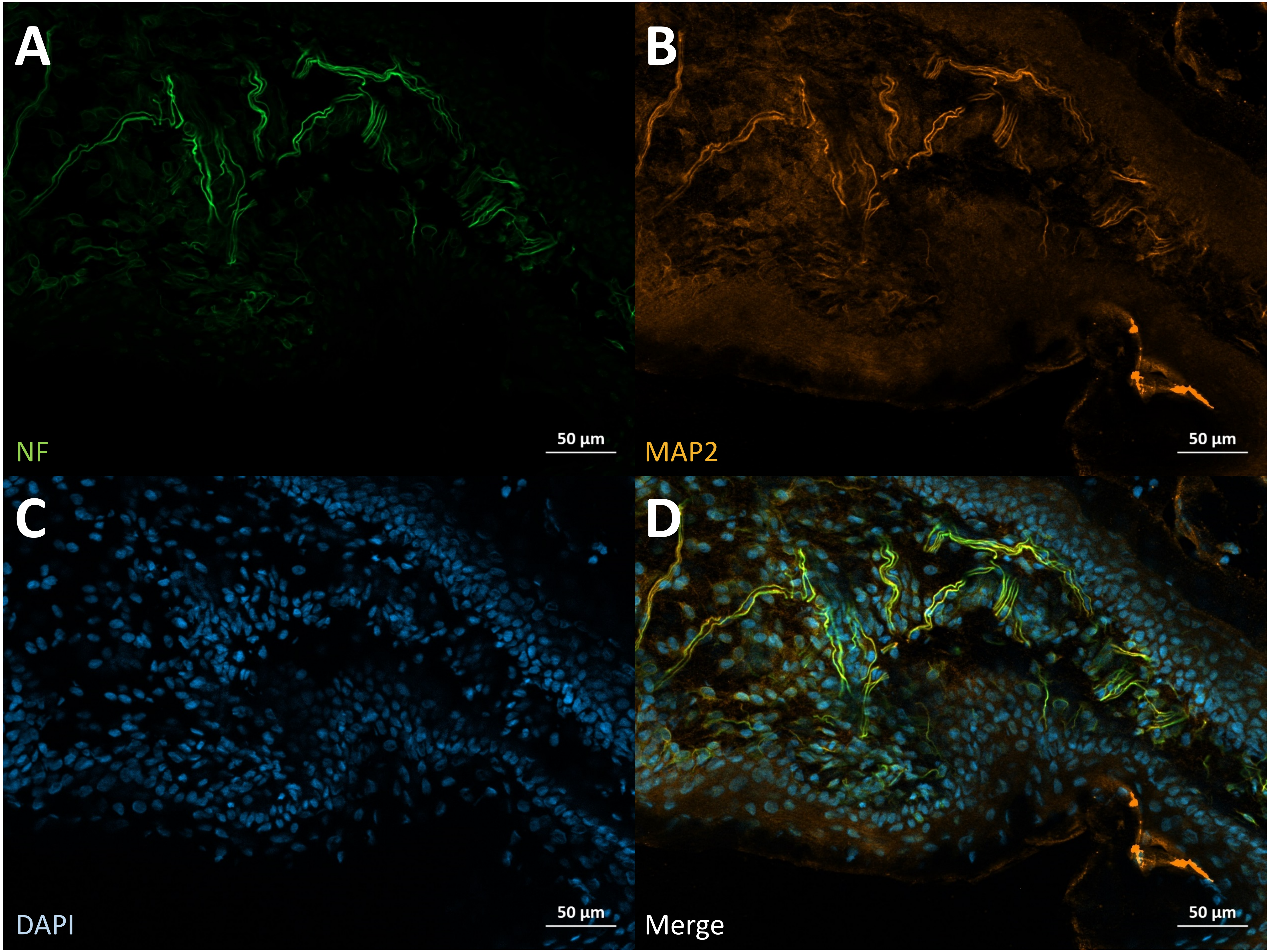

Supplementary figure f

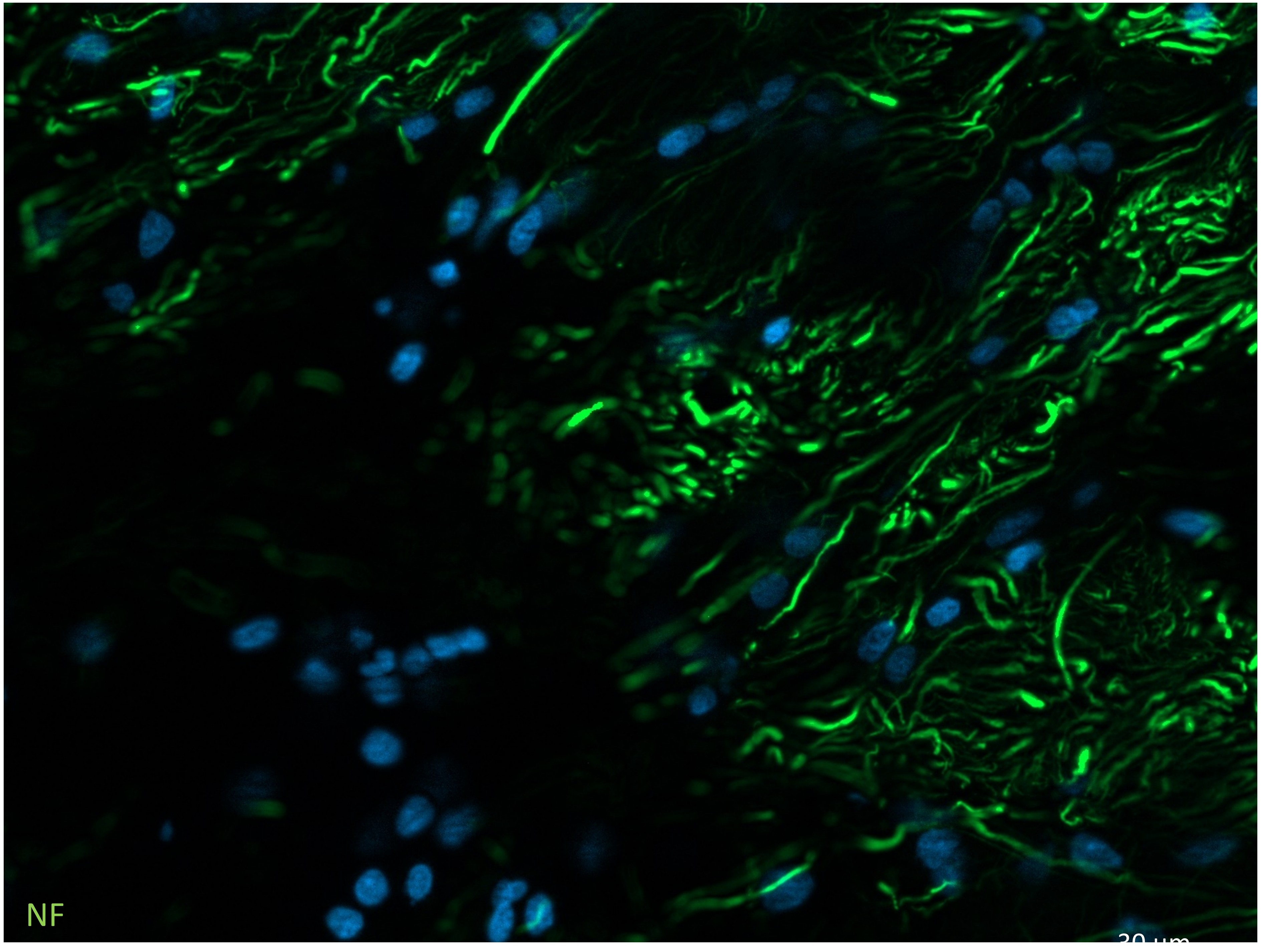

Supplementary figure g

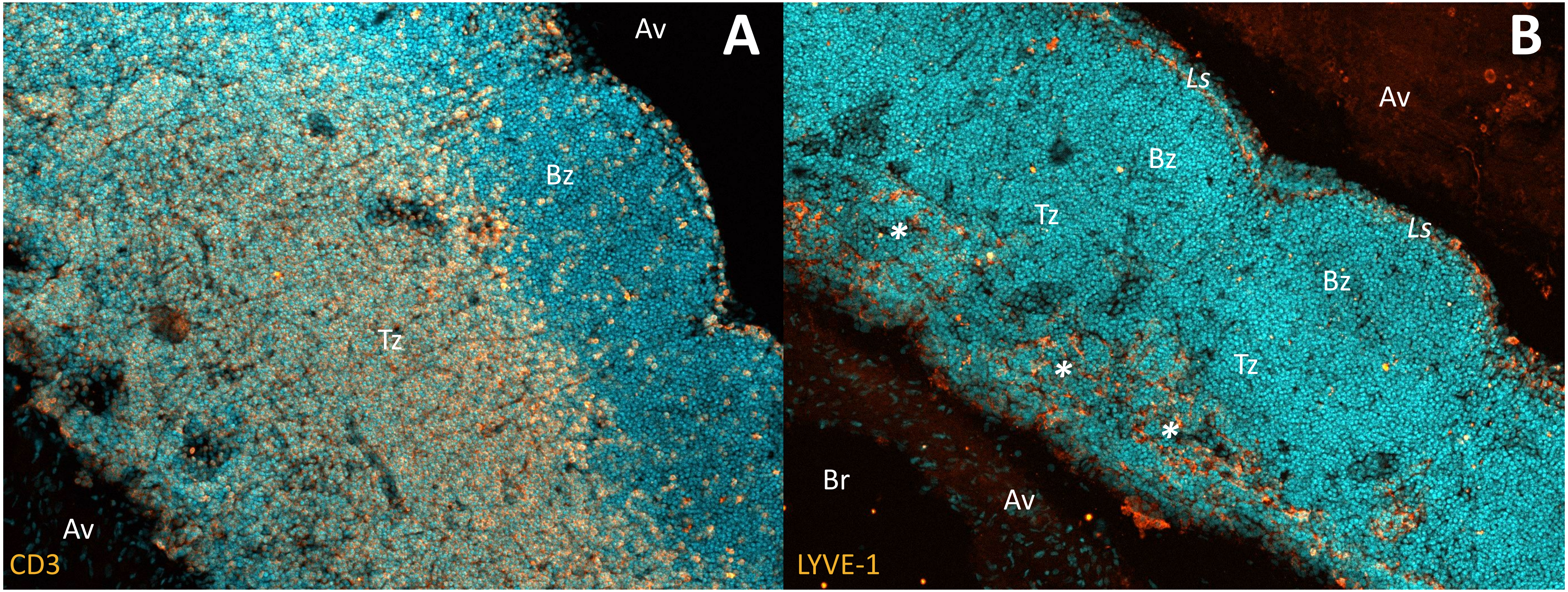

Supplementary figure h

Supplementary - table 1: statistical information about species, number of organs, slices and type of section

Organ / tissue	samples total	slices each	slices total	species	serial section
thymus	4	150	600	rat	yes
spleen	4	100	400	rat	yes
dermis	4	30	120	rat	no
NALT	3	30	90	mouse	yes
BALT	3	30	90	rat	yes
Peyer‘s patches	4	30	120	rat	yes
brain	2	30	60	rat	no
brain	2	30	60	mouse	no
bone marrow (femur)	2	20	40	rat	no
superficial cervical lymph node	4	30	120	rat	yes
facial lymph node	4	30	120	rat	yes
brachial lymph node	4	30	120	rat	yes
axillary lymph node	4	30	120	rat	yes
superior mesenteric lymph node	4	30	120	rat	yes
inguinal lymph node	4	30	120	rat	yes
popliteal lymph node	4	30	120	rat	yes
renal lymph node	4	30	120	rat	yes
